# The Montreal Protocol and the fate of environmental plastic debris

**DOI:** 10.1007/s43630-023-00372-x

**Published:** 2023-01-27

**Authors:** M. A. K. Jansen, P. W. Barnes, J. F. Bornman, K. C. Rose, S. Madronich, C. C. White, R. G. Zepp, A. L. Andrady

**Affiliations:** 1 School of Biological, Earth and Environmental Sciences, Environmental Research Institute, University College Cork, Cork, Ireland; 2 Biological Sciences and Environmental Program, Loyola University New Orleans, New Orleans, LA USA; 3 Food Futures Institute, Murdoch University, Perth, Australia; 4 Biological Sciences, Rensselaer Polytechnic Institute, Troy, USA; 5 Atmospheric Chemistry Observations and Modeling Laboratory, National Center for Atmospheric Research, Boulder, CO USA; 6 Exponent, Inc, Bowie, MD 20715 USA; 7 ORD/CEMM, US Environmental Protection Agency, Athens, GA USA; 8 Chemical and Biomolecular Engineering, North Carolina State University, Raleigh, NC USA

## Abstract

Microplastics (MPs) are an emerging class of pollutants in air, soil and especially in all aquatic environments. Secondary MPs are generated in the environment during fragmentation of especially photo-oxidised plastic litter. Photo-oxidation is mediated primarily by solar UV radiation. The implementation of the Montreal Protocol and its Amendments, which have resulted in controlling the tropospheric UV-B (280–315 nm) radiation load, is therefore pertinent to the fate of environmental plastic debris. Due to the Montreal Protocol high amounts of solar UV-B radiation at the Earth’s surface have been avoided, retarding the oxidative fragmentation of plastic debris, leading to a slower generation and accumulation of MPs in the environment. Quantifying the impact of the Montreal Protocol in reducing the abundance of MPs in the environment, however, is complicated as the role of potential mechanical fragmentation of plastics under environmental mechanical stresses is poorly understood.

## Introduction

Plastic debris in the environment is an increasing pollution problem, and a multitude of studies has convincingly demonstrated the ubiquity of plastic debris, including microplastic (MP) particles, across planet Earth^1^. An estimated 8300 million metric tonnes of plastics have been produced since the 1950s, of which ca 80% has ended in landfills and the natural environment [1]. As of 2016, ca. 19–23 million metric tonnes per year, or 11% of all plastic waste generated, was estimated to have entered aquatic ecosystems [2]. Polyethylene (PE), polypropylene (PP), polystyrene (PS), and poly(ethylene terephthalate) (PET) account for ca. 70% of all MPs in freshwater ecosystems [3]. An estimated 11.6–21.1 million tonnes of MPs made of PE, PP and PS occur in the top 200 m of the Atlantic Ocean [4]. Concerns about potential risks posed by MPs to the environment and human health have prompted much research. There are also calls for a global treaty on plastics towards a more sustainable future [5].

Breakdown of plastics occurs due to abiotic and biotic factors [6]. Micro- and nanoplastics (typically defined as plastic particles < 5 mm, and < 0.1 µm in size, respectively (but see [7]) are generated in the natural environment as a result of solar ultraviolet (UV)-driven weathering of plastic debris in combination with fragmentation due to exposure to mechanical forces [6]. These micro- and nanoplastics are widely distributed in aquatic and terrestrial ecosystems and also pose a potential risk to humans through inhalation [8], ingestion [9] and dermal contact [10]. MPs have been found, for example, in bottled drinking water [11], table salt [12], and seafood [13]. A recent estimate [9] places the annual human intake of MPs from all sources to be 10^5^ particles. Small MPs (1–5 µm) may enter systemic circulation and translocate into cellular compartments [14, 15].

Recently, MPs in human placenta have been detected in studies carried out in clinical settings. In one of these, PP particles, 5–10 µm in size, were found in placenta samples from vaginal deliveries [16]. A second study detected even larger MPs > 50 µm of PE, PS, and PP in human placenta and meconium from caesarean delivery [17], where the chance of contamination via the birth canal is excluded. Although some of the MPs crossed the placental barrier into the foetal side, no foetal translocation was noted, unlike in studies on inhaled MPs in rats where foetal translocation was observed [18]. Despite these concerning findings, negative human physiological impacts of micro- and nanoplastics have not been conclusively established [19].

Assuming current trends in global production of plastics, and no improvements in waste management infrastructure worldwide, releases into the environment may grow to 90 million metric tonnes per year by 2030 [2]. Given the recalcitrance of plastics to environmental degradation as well as potential negative biological and health impacts [20], there is particular concern about the risks posed by micro- and nanoplastic particles and similarly sized plastic fibres in terrestrial and aquatic ecosystems globally.

This current assessment focuses on the interactive effects of solar radiation, its UV component, and climate change on the fate of environmental plastic debris, with regard to degradation and fragmentation and their potential consequences. This assessment is part of the journal issue of the Quadrennial Assessment by the Environmental Effects Assessment Panel (EEAP) of the Montreal Protocol under the United Nations Environment Programme (UNEP).

## Photo-oxidation and plastic persistence

**Fig. 1:**
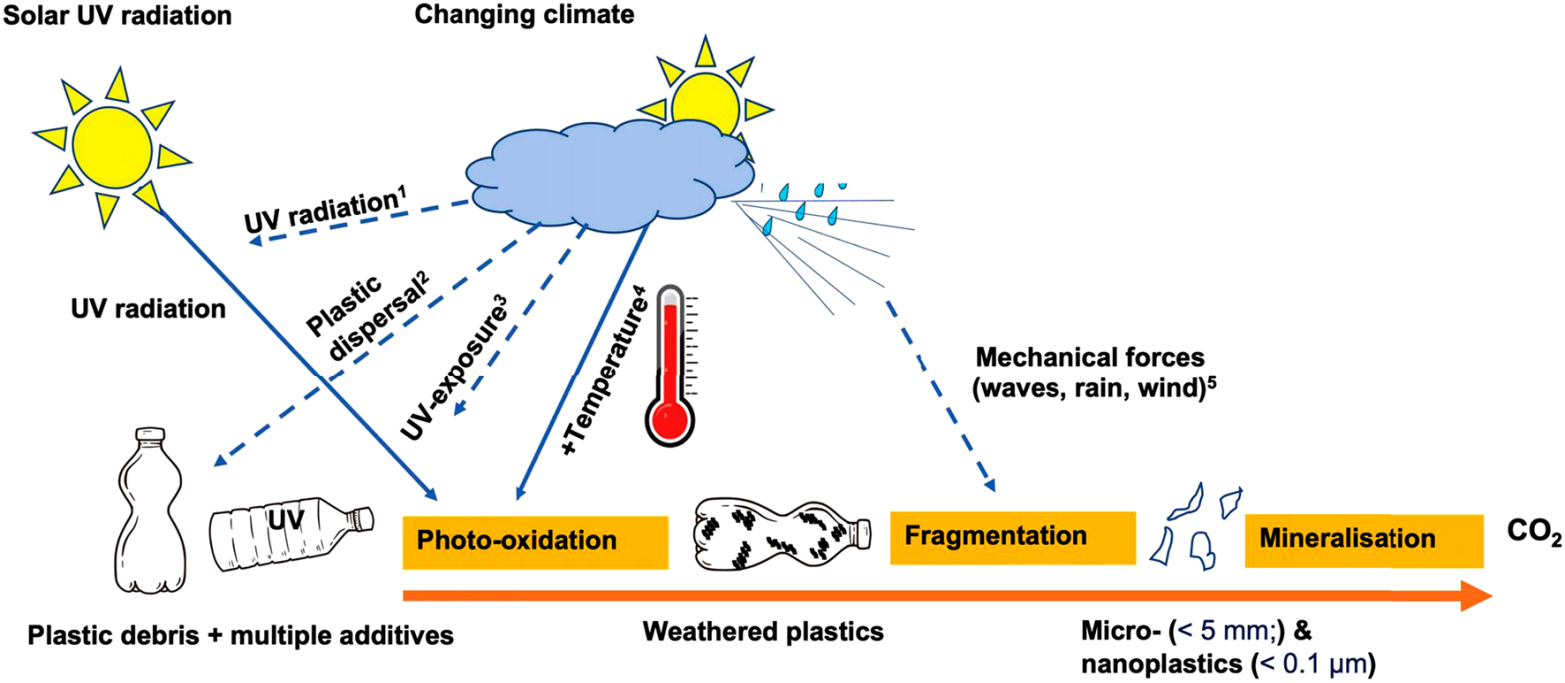
Solar UV radiation can drive the photo-oxidation of plastics, making plastics prone to fragmentation, a process that may result in the formation of microplastic particles. Plastic mineralisation has been reported, but the relevance of this process in the natural environment remains to be established. The climate impacts on photo-oxidation through a variety of different routes, including (1) direct effects on solar UV radiation; (2) plastic dispersal; (3) altered penetration of UV radiation through the water column; and (4) increased local temperatures. Climate may also impact the fragmentation of weathered plastics by (5) affecting mechanical stress fields

A major barrier towards a realistic assessment of the global impacts of plastics is the incomplete knowledge of the fate, and particularly the degradation and fragmentation of plastics in the environment [21]. Exposure to solar UV radiation is the primary weathering mechanism of plastics debris (Fig. 1), making plastics prone to subsequent fragmentation into smaller particles [20, 22–26]. Photo-oxidation of plastic debris under extended outdoor exposure makes the material weak, brittle and prone to subsequent fragmentation [26, 27]. Fragmentation occurs when plastics are subjected to, for example, wave action or encounters with animals, resulting in the generation of secondary micro- or nano-particles^2^ (Fig. 1). MPs sampled from beach and surface water environments show spectroscopic signatures of photo-oxidation, primarily the presence of surface carbonyl groups [28, 29], as well as increased fractional crystallinity [30]. While UV irradiation drives photo-oxidation, and therefore contributes to the fragmentation of plastic debris into progressively smaller sizes, it may also help remove plastic particles from the environment through photo-mineralisation [21, 27, 31]. There is evidence from laboratory-accelerated approaches that MPs can undergo UV-induced mineralisation into carbon dioxide (CO_2_) and water [21, 31] (Fig. 1). However, the phenomenon has not been conclusively shown to occur in natural environments, and if it does occur in nature, only a small fraction of the already highly fragmented plastics with a large specific surface area, is likely to be involved.

## Different plastics and photo-oxidation

In addition to base polymers, plastics generally contain catalyst residues and unreacted monomers, as well as intentionally added chemicals including plasticisers, dyes, antioxidants, flame retardants and/or UV stabilisers [32, 33]. Said mixture has a considerable impact on the rate of photo-oxidation and subsequent fragmentation of plastics. For example, high-density PE and nylon-6 plastics generate MPs when exposed to the equivalent of 44 days of solar irradiation, whereas high-impact PS and PP did not [34]. It remains to be determined whether differences in photo-oxidation relate to the base polymer, or rather specific additives. There is a substantial knowledge gap concerning action spectra of photo-oxidation, and dose–response curves, in the context of the composite characteristics of commercial plastics. Further, laboratory studies have shown that UV-associated degradation rates in simulated aquatic conditions are further mediated by other environmental factors, including temperature, oxygen availability and salinity [35].

## The Montreal Protocol and photo-oxidation

The anticipated significant increase (“the World avoided”) in terrestrial solar UV radiation, avoided by the implementation of the Montreal Protocol and its Amendments, would have increased the rates of photodegradation, and consequently fragmentation of plastic debris. It is currently not known whether a critical threshold of photo-oxidation for a given plastic is required to facilitate fragmentation. This presents a significant gap in knowledge. Also, little is known about the quantitative mechanical forces required to cause fragmentation, and how this force requirement is affected by the photo-oxidation state. Even non-oxidised plastics can be fragmented if mechanical forces are large enough [36]. However, how these forces compare with naturally occurring stress-fields has not been well studied. There is evidence that virgin plastics can be fragmented in the gut of ingesting crustaceans [37, 38]; similarly, MPs can be generated as a consequence of the mechanical forces imposed on objects as diverse as car tyres [39] or artificial sports turf [40]. Thus, at this stage the relative importance of solar radiation, and weathering of plastics in facilitating fragmentation is not clear.

## Plastic degradation and UV radiation in a changing climate

Both stratospheric ozone depletion and climate change can alter the irradiance of solar UV radiation reaching the Earth’s surface [41], thus affecting photo-oxidation of plastics. Locally, strong increases in temperature under future climate scenarios may further accelerate the rate of photo-oxidation leading to fragmentation (Fig. 1). At present, there are very significant gaps in knowledge pertaining to the impact of global changes on plastic persistence. Increased temperature consequent to climate change is not the only factor that may affect the rate of plastic degradation. For example, increased stress-fields in aquatic environments cause fragmentation, changes in relative humidity alter photodegradation rates, sedimentation rates affect biodegradation, and increased rainfall patterns that control runoff have an effect on plastic dispersal, vertical mixing and transparency of aquatic ecosystems [42] (Fig. 1). Conversely, plastics also affect climate change by being a significant sink of global carbon [43]. Other, more subtle impacts of MPs will affect carbon storage. For example, ingestion of MPs by a zooplankton species, *Salpa fusiformis* (also known as the common salp), increases the buoyancy of faecal pellets thereby decreasing downward transport and burial of marine carbon in a process called the “biological pump” [44]. Projected future increases in marine MP concentrations may thus reduce carbon sinking rates in the oceans, and therefore alter ocean carbon cycling [44]. Thus, there is a myriad of poorly detailed interactions between UV radiation, global change and plastics, affecting, amongst others, the fate of plastics in the natural environment.

## Exposure of environmental plastic debris to UV radiation

To quantify the environmental rate of UV-driven photodegradation, it is necessary to evaluate the dispersal and distribution, i.e. exposure to UV radiation, of plastic debris [31, 45]. Especially significant from a UV-exposure perspective are air-borne, floating and beach debris. Airborne particles are dominated by fibres, including microplastic fibres [46]. Smaller plastic particles, including abrasive tyre wear^3^ [47], may remain air-borne for weeks [48, 49], and this is associated with strong UV irradiance. In the terrestrial environment there has been a rapid growth in the use of plastics in agricultural systems, for example, the use of plastic mulch to reduce weed growth and maintain optimal soil moisture and temperature. Such applications are associated with exposure to UV radiation, and fragments may enter the atmosphere and reach remote ecosystems [47, 50]. Conversely, other uses of plastics such as soil improvement using polyurethane foam [22] will not typically result in exposure to UV radiation of the plastics.

In the aquatic environment, exposure to UV radiation depends strongly on buoyancy, although advective water flow and turbulence results in sedimentation of larger numbers of MPs than would be expected from gravitational sedimentation alone in both the freshwater [51] and marine environments [52]. In the oceans, the global mass of floating plastic debris represents only a small percentage of the estimated annual influx of plastics into the aquatic environment, based on production volumes [43, 45, 53, 54], and only these floating plastics will experience UV irradiation. In contrast, sedimentation of plastics will minimise exposure to UV radiation. Sedimentation is linked to geometric and other physical properties of marine MPs, as well as biofouling [55], i.e. the development of a surface layer of microorganisms, algae, and small shelled species on the plastics. This increases the density of plastic debris [4, 56] driving sedimentation. Nevertheless, sedimentation is far from a one-way process. In a well-mixed ocean, biofouled MPs can oscillate vertically in the water column, with the depth of the oscillation depending on, for example, algal growth and light penetration [57]. Still, the nett, long-term sedimentation removes plastics from the photic zone, thus slowing down photo-oxidation.

## Biological consequences of photo-oxidation and fragmentation

UV radiation-driven photo-oxidation of plastic debris, and subsequent fragmentation following exposure to mechanical forces, will alter the size distribution of plastics in the natural environment. However, the quantification of the UV-mediated changes in this plastic size distribution is lacking. In fact, a major deficiency in the study of the biological impacts of all plastics in the environment is the lack of reliable, quantitative knowledge of environmentally relevant concentrations of micro- and nanoplastics in different environments. This, in turn, relates to a lack of adequate, and standardised, monitoring technology, particularly in complex matrices such as, for example, soil [58]. Concentrations of larger MPs are best known. For example, Sembiring et al. [59] estimated MP (> 125 µm) concentrations in an Indonesian river and the downstream seawater to be 0.06 and 3000 particles/m^3^, respectively. The average MP concentration in river sediment was 16.7 particles/100 g and in marine sediment 3.3 particles/100 g. However, such numbers may vary considerably depending on the sampling and monitoring approach, as well as the actual location [60].

At present there is a lack of quantitative information on the presence of nano- and smaller microplastics in diverse environments. Given that it has been speculated that nano- and smaller microplastics will have a greater impact on organisms than larger plastics as a result of their transport properties, bioavailability, relative surface area and scope for additive leaching, ingestion and/or uptake in cells [61], this does hamper the assessments of risks associated with plastic pollution.

Both hazards and risks associated with MPs have been analysed and reported, although at present much uncertainty remains concerning biological impacts under realistic environmental concentrations of plastics. Large research gaps exist in the quantitative analysis of the relationship between various exposure routes of MPs, and the actual measured MP or NP toxicity [60]. For example, as the distribution of MPs in the environment is heterogeneous, different organisms will be exposed to different plastics. For example, PE and PP will (initially) float, while plastics such as poly(vinyl chloride) (PVC) sink more readily and as a consequence, organisms with different feeding habits will be differently exposed. Publication bias is also of some concern, with results showing a lack of biological impacts less likely to be published [62]. Nevertheless, and despite above mentioned reservations, research shows that plastics can potentially exert significant negative impacts on selected species of a very broad range of marine, freshwater and terrestrial species [63]. However, other studies fail to observe significant negative impact [62, 64]. This apparent lack of consistency across large numbers of studies suggests that experimental conditions, including MP concentration, size, shape and composition as well as the chosen test organism all play a role in the different outcomes of toxicity [64].

Historically, toxicological studies have predominantly focussed on marine taxa with relatively small sized organisms [3], with less data on the impacts of MPs on large animals, at high trophic levels or terrestrial biota [10]. Effects of MPs on plants and ecosystem productivity remain uncertain [20, 65–67]. Marine studies have indicated that zooplankton are more affected by plastics than many other taxa, with obvious consequences for the entire food web. The transfer of plastics up the food chain from primary producers to consumers is also of some concern [68, 69], although evidence of accumulation at higher trophic levels remains limited at present. Finally, understanding of the exposure to and uptake and effects of various types (synthetic, semi-synthetic or natural) of anthropogenic fibres is still in its infancy, notwithstanding the ubiquitous presence of these fibres in the natural environment [70].

A particular difficulty in exposure studies is the fact that plastics are a complex material comprised of different polymers, stabilisers, dyes and other additives. Many of these additives can leach out and exert toxic effects in their own right [32]. Thus, the same plastic base material may exert different toxic effects depending on the additives used in them. UV radiation may drive photo-oxidation, and ultimately photofragmentation, leading to increasing numbers of MPs with increased fragment surface area. This, in turn, can stimulate the leaching of plastic additives, such as endocrine disrupting chemicals that adversely affect organisms [71]. Plastic leachates activate oxidative stress responses in cell-based bioassays [72]. However, low environmental concentrations of leached chemicals indicate that effects in the natural environment may be limited [3]. UV radiation-driven photo-oxidation of plastic surface area can also decrease binding capacity for some organic substances [73], although increased absorptive capacity of plastics towards substances such as the antibiotic ciprofloxacin and the endocrine disruptor bisphenol-A has been reported [74]. Similarly, prior exposure to UV radiation can increase the binding capacity of plastics for heavy metals [75, 76].

Overall, a substantial knowledge gap remains concerning the effects of UV-mediated photo-oxidation and fragmentation, with expected impacts on size distribution of environmental plastics, as well as additive leaching and contaminant binding, all of which are likely to depend on plastic type, duration of exposure, and contaminant chemistry [25, 26, 77].

## Knowledge gaps

The links between UV irradiation, the stratospheric ozone layer, and MP pollution, although highly relevant, are still poorly understood and scarcely addressed by the scientific community working on MPs. Major knowledge gaps relate to environmental distribution of plastics, and consequent exposure to UV radiation. While it is recognised that some plastics will be buried in sediments where penetration of UV radiation will be virtually nil, others will be air-borne and potentially exposed to considerable amounts of UV radiation. Furthermore, where plastics are exposed to UV radiation, uncertainties about the UV dose–response of photo-oxidative reactions impede assessments of weathering and subsequent fragmentation. Thus, while UV-driven photo-oxidation of plastics, and subsequent fragmentation are well known, the quantitative impact of these processes on plastic longevity and MP generation remains unknown.

## In conclusion

UV-driven weathering, followed by subsequent fragmentation can lead to a decrease in plastic macro-debris in the environment, yet increase the concentration of MPs. By integrating existing surface UV irradiation data with better knowledge of the distribution of plastics across various environmental niches, there is an opportunity to generate quantitative predictions of plastic persistence at a global scale. In turn, such insights can inform the design of more environmentally friendly plastics. However, this approach will require better knowledge of action spectra and dose–response relationships of UV-driven oxidation of common compounded plastics, which include intentionally added chemicals such as plasticisers, dyes, antioxidants, flame retardants and/or UV stabilisers [32, 33]. It is also recognised that quantitative predictions of plastic persistence will be subject to effects of climate change, which may affect processes as diverse as the penetration of UV radiation into the water column, sedimentation rates and/or air movements. Furthermore, UV irradiation can also affect the chemical or toxicological properties of MPs and may play a key role in determining hazards and risks associated with MPs. Therefore, there is an urgent need to better understand the interactions between plastics in the environment, climate change, and UV radiation.

## Relevance to the sustainable development goals

The Montreal Protocol and its Amendments contribute to several of the United Nations Sustainable Development Goals (SDG) through protection of the stratospheric ozone layer and the mitigation of climate change. SDG targets addressed in this section are detailed below.

### SDG 6: clear water and sanitation

There is ample evidence that MPs are ubiquitous in freshwater and marine environments. Consequently, essential products such as drinking water can be contaminated by MPs. The implementation of the Montreal Protocol has resulted in the avoidance of high UV irradiation, which is a key driver of plastic weathering, and ultimately, generation of MPs.

### SDG 14: life below water

Macro-, micro,- and nanoplastic pollutants are ubiquitous in freshwater and marine environments. Consequently, aquatic organisms and ecosystems are exposed to these man-made pollutants. The hazardous character of MPs to aquatic organisms has been shown in some studies, although ecological risks remain to be established. The implementation of the Montreal Protocol has resulted in the avoidance of high UV irradiation, and this is likely to have resulted in decreased weathering, and ultimately, decreased generation of MPs. Conversely, implementation of the Montreal Protocol is likely to have resulted in increased persistence of macroplastic debris, which has been widely shown to have negative impacts on animals due to entanglement or accumulation in, for example, the stomach.

### SDG 15: life on land

Climate change is impacting agricultural practices and has, amongst others, been associated with the increased use of plastics in farming. In turn, this may result in the accumulation of an appreciable plastic burden in agricultural soils with consequences for soil biochemistry, including soil microbiology and nitrogen cycling. The implementation of the Montreal Protocol has led to the avoidance of high UV irradiation but this, in turn, can extend plastic longevity and lead to land degradation and soil biodiversity loss.

## Data Availability

All data generated or analysed are included.

## Footnotes

1 This Perspective is part of the topical collection: Environmental effects of stratospheric ozone depletion, UV radiation, and interactions with climate change: UNEP Environmental Effects Assessment Panel, 2022 Quadrennial Assessment.

2 Microplastics is a misnomer as it is generally taken to mean all plastic fragments < 5 mm in dimension. Nanoplastics has been used, depending on the publication, to mean MPs that are < 1000 nm or 100 nm.

3 A wide range of micro-sized particles in the environment are either non-plastic, or part-plastic anthropogenic particles, including tyre wear, paint particles and fibres. For example, tyres are made of elastomeric polymers (or rubber) and are not thermoplastic but thermosets. However, they have been generally included in the category ‘microplastics’ along with other thermosets such as polyurethane foam and epoxy.
